# Electroactive Imidazolium Ionic Liquids in Organic Synthesis

**DOI:** 10.1021/acs.accounts.6c00302

**Published:** 2026-06-29

**Authors:** Cristiana Margarita, Fabrizio Vetica, Marta Feroci

**Affiliations:** † Department of Basic and Applied Sciences for Engineering (SBAI), 9311 Sapienza University of Rome, via Castro Laurenziano 7, 00161 Rome, Italy; ‡ Department of Chemistry, Sapienza University of Rome, Piazzale Aldo Moro 5, 00185 Rome, Italy

## Abstract

The renewed interest in electrochemistry in organic synthesis has provided new opportunities for the development of novel and efficient chemical transformations. In this context, ionic liquids (ILs) have emerged as privileged media due to their peculiar properties, including negligible vapor pressure, high coordination ability and thermal stability, and a wide electrochemical window. Among them, imidazolium-based ILs are of high interest since they can be used not only as solvent/supporting electrolytes but also as electrochemical precursors for reactive species. This Account discusses recent advances in the use of electroactive imidazolium-based ILs in organic synthesis, focusing on the electrogeneration of Lewis acids (LAs) and N-heterocyclic carbenes (NHCs). Key strategies are discussed, with a critical discussion of the electrochemical behavior and setups as well as their impact on reaction design and selectivity as compared with conventional synthetic methods. Overall, this work aims to provide a comprehensive overview of the emerging possibilities that the combination of electrochemistry and ionic liquids provides and to outline the possible future perspectives for their applications in organic synthesis.

## Graphical abstract

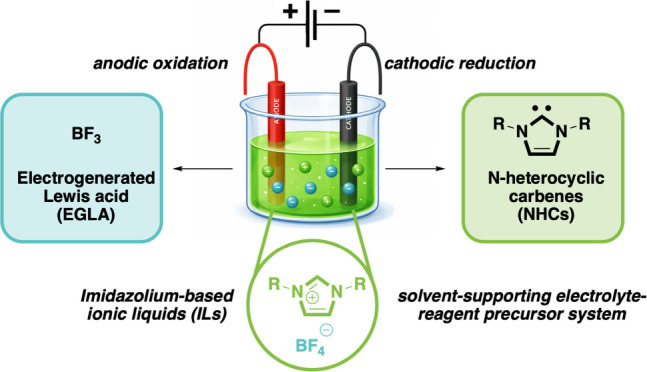

## Key References

-

Bortolami, M. ; Magboo, F. J. P. ; Petrucci, R. ; Vetica, F. ; Zollo, G. ; Feroci, M. Electrogenerated BF_3_ From Tetrafluoroborate-Based Ionic Liquids: Theoretical And Experimental Studies Towards Selective Styrene Oxide Isomerization. J. Electrochem. Soc. 2021, 168­(11), 115501 10.1149/1945-7111/ac39e2 .^1^ This paper reports an experimental and theoretical study on the stabilizing effect of the BMIm-BF_4_ anion and cation on electrogenerated BF_3_ and puts in evidence the higher stability of BF_3_ in IL with respect to BF_3_ etherate.
-

Bortolami, M. ; Mattiello, L. ; Scarano, V. ; Vetica, F. ; Feroci, M. In Situ Anodically Oxidized BMIm-BF_4_: A Safe and Recyclable BF_3_ Source. J. Org. Chem. 2021, 86, 16151–16157 10.1021/acs.joc.1c00932 34213898 PMC8609525.^2^ This paper demonstrates the efficiency of electrogenerated BF_3_ use in four classical Lewis acid-catalyzed organic reactions, obtaining similar or improved yields as those obtained using BF_3_ etherate, with the possibility to efficiently recycle the used IL.
-

Rocco, D. ; Folgueiras-Amador, A. A. ; Brown, R. C. D. ; Feroci, M. First example of organocatalysis by cathodic *N*-heterocyclic carbene generation and accumulation using a divided electrochemical flow cell. Beil. J. Org. Chem. 2022, 18, 979–990 10.3762/bjoc.18.98 PMC935920235965857.^3^ This work demonstrates the possibility to electrogenerate and use NHCs under continuous flow conditions in a divided cell, with the chance of IL recycling. A batch vs flow electrolysis comparison is reported.
-

David, M. ; Galli, E. ; Brown, R. C. D. ; Feroci, M. ; Vetica, F. ; Bortolami, M. 1-Butyl-3-methylimidazolium tetrafluoroborate as suitable solvent for BF3: the case of alkyne hydration. Chemistry vs electrochemistry. Beil. J. Org. Chem. 2023, 19, 1966–1981 10.3762/bjoc.19.147 PMC1076048438169890.^4^ In this paper, a comparison between the electrochemical and the conventional chemical approach in BF_3_-catalyzed alkyne hydration in IL is reported. The possibility of using BF_3_ from less hazardous precursors is demonstrated.

## Introduction

Electrochemistry has recently re-emerged as a powerful and sustainable tool in organic synthesis, offering unique opportunities to access innovative reactivities and pathways that are often difficult to achieve under conventional reaction conditions. By directly employing electrons as reagents, electrochemical approaches enable precise control over redox processes without the need for stoichiometric amounts of oxidants and reductants. This renewed interest is driven by the increasing demand for green and sustainable synthetic methodologies, paired with technological advancements that made electrochemical setups available and easily accessible. Hence, in the past decade, the scope and applicability of electro-organic transformation have been radically broadened.^5–8^

In parallel, ionic liquids (ILs) have attracted the attention of the scientific community as alternative and sustainable solvent systems in organic synthesis.^9–11^ These compounds, typically composed of an organic cation and different organic or inorganic counteranions, are characterized by neglectable vapor pressure, high thermal stability, enhanced solvation properties (specifically for ionic intermediates), and wide electrochemical windows. Moreover, their properties are easily tunable, affecting polarity, viscosity, and coordinating ability, thus making them particularly attractive as versatile reaction environments.^12^

The combination of ionic liquids and electrochemistry represents a promising platform to achieve novel, efficient, and sustainable synthetic methods. In fact, the ionic nature of ILs paired with wide electrochemical windows makes them excellent solvents for electrochemical transformations, since ILs themselves can serve as both solvents and supporting electrolytes, hence reducing chemical waste and avoiding the use of additional salts to guarantee conductivity.^13^ Within this context, imidazolium-based ionic liquids have been widely studied in electro-organic synthesis, not only as reaction media but also as reactive intermediate precursors. Their intrinsic reactivity under electrochemical conditions can be effectively harnessed for catalytic transformations. Notably, these ionic-liquid-derived active species can be generated prior to the catalytic step, while the highly coordinating environment provides their efficient stabilization, allowing their subsequent use in a controlled manner. This strategy minimizes undesired electrochemical side reactions and provides accurate regulation of both reactivity and selectivity.

In this Account, we describe the applications of electroactive imidazolium-based ionic liquids as solvents, supporting electrolytes, and reactive species precursors in organic synthesis. Specifically, the possibility to electrogenerate Lewis acids (LAs) or *N*-heterocyclic carbenes (NHCs) starting from these ILs is discussed, focusing on their electrochemical behavior and the electrochemical setups and reaction conditions and how these influence the synthetic outcomes of the methodologies.

## Electroactive Ionic Liquids as Lewis Acid Precursors

The electrochemical generation of Lewis acids (LAs) in conventional organic media represents a powerful strategy to access reactive species under mild conditions. This approach typically relies on anodic dissolution or oxidation processes that generate metal cations or electrophilic intermediates (Figure 1a). Electrogenerated Lewis acids (EGLA) can then be used as catalysts to promote organic transformations. A representative example is the indirect electrogeneration of BF_3_ from tetrafluoroborate (BF_4_^–^).^14^ In this system, anodic oxidation of a titanium electrode, using methanol as solvent, produces Ti^4+^ species. By employing an undivided cell configuration and in the presence of NaBF_4_ as a supporting electrolyte, Ti^4+^ ions can abstract fluoride anions from BF_4_^–^ to generate BF_3_. The resulting LA is immediately exploited in situ to promote the esterification between methanol and formic acid (the latter obtained via concurrent electrochemical CO_2_ reduction), affording methyl formate in an integrated electrochemical process. This electrochemical system allowed for the efficient production of methyl formate with a high faradaic efficiency of 69%, observed at a current density of 5.3 mA cm^–2^. This example highlights the ability of electrochemical methods to couple catalyst generation and substrate transformation within a single setup, thereby avoiding the direct handling of corrosive BF_3_. In a similar approach, Wen, Guo, and co-workers reported the anodic oxidation of aluminum electrodes, leading to the formation of Al^3+^ species, which in the presence of chloride ions generate AlCl_3_. This EGLA efficiently promotes the reduction of sulfoxides to sulfides under relatively mild conditions, even up to the multigram scale.^15^ The use of sacrificial electrodes thus provides a convenient route to classical Lewis acids without requiring their external addition.

**Figure 1:**
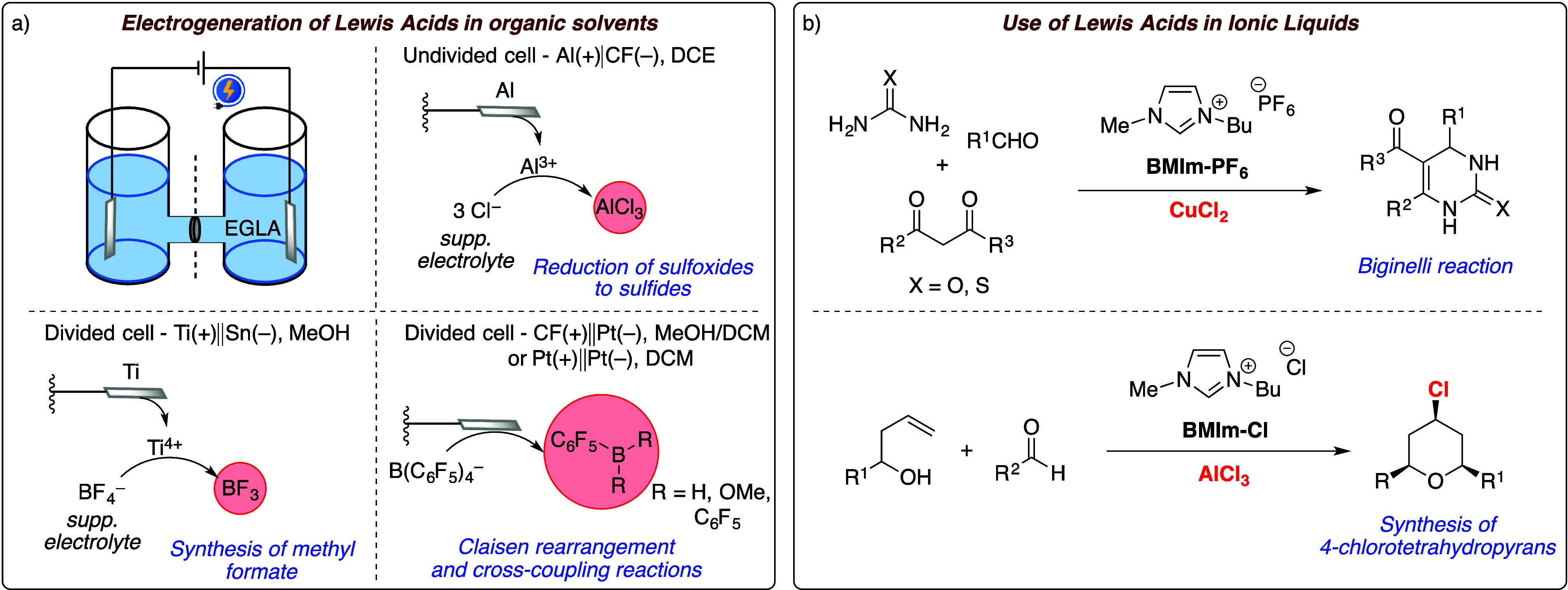
(a) Electrogeneration of LAs or (b) the use of LAs in ILs.

More recent developments have expanded this concept to non-metal-centered LAs. For instance, the anodic oxidation of tetrakis­(penta­fluorophenyl)­borate leads to the formation of highly electrophilic boron species capable of promoting defluorination reactions.^16^ Akiba et al. demonstrated the synthetic applicability of this approach, leveraging the reactivity of a naked boron LA species, for defluorinative cross-coupling and alkene metathesis reactions. In a divided cell setup and upon electrochemical treatment, the authors could verify by NMR analyses the formation of bis­(perfluoro­phenyl)­borane as an active Lewis acid.

Afterward, similar EGLA acids have been successfully applied in Claisen rearrangements, demonstrating the versatility of this approach in complex bond-forming reactions.^17^ The authors performed a detailed study of the electrogeneration of LA using a mixture of MeOH and DCM as the solvent. ^1^H and ^11^B NMR analyses of the anolyte solution highlighted the formation of MeOH-derived boron adducts, specifically B­(C_6_F_5_)_3_, B­(OMe)­(C_6_F_5_)_2_, and B­(OMe)_2_(C_6_F_5_). The formation of boron–solvent adducts decreases the Lewis acidity of the system, which, however, can be employed to tune the reactivity of highly reactive substrates, suppressing the formation of side products. In fact, the intended Claisen rearrangement reported by the authors required extremely short reaction times (from 1 to 6 min) using DCM/MeOH as a solvent.

Overall, these examples underline the key advantages of LA electrogeneration in organic solvents: (i) temporal control over Lewis acid formation, (ii) avoidance of stoichiometric reagents, and (iii) compatibility with tandem or cascade processes.

In contrast to electrogeneration strategies, ionic liquids (ILs) have also been employed as noninnocent solvents capable of stabilizing and enhancing the activity of classical LAs. The unique solvation environment provided by ILs enables the formation of stable Lewis acid–anion complexes, often leading to improved catalytic performance (Figure 1b).

A seminal example is the use of chloroaluminate ILs, such as BMIm-Cl/AlCl_3_ systems, for the synthesis of 4-chlorotetrahydropyrans.^18^ In this system, the ionic liquid not only serves as a solvent but also participates in the formation of active LA species through equilibria between AlCl_3_ and chloride ions. Similarly, the Biginelli reaction has been efficiently performed in ionic liquids in the presence of Lewis acids such as CuCl_2_.^19^ The IL environment stabilizes charged intermediates and facilitates multicomponent condensation reactions, often leading to improved yields and selectivities compared to those of conventional solvents. More recently, ionic liquids have also been employed in polymerization reactions, such as the FeCl_3_-catalyzed polymerization of β-pinene.^20^

A major conceptual advance in this field is the use of ionic liquids not only as solvents but also as intrinsic precursors for the electrogeneration of Lewis acids, leading to integrated solvent–catalyst systems and thus combining the advantages of the two approaches just discussed. This strategy is exemplified by tetrafluoroborate-based imidazolium ionic liquids (e.g., BMIm-BF_4_), as shown in Figure 2. In the first key study, anodic oxidation of the BF_4_^–^ anion in BMIm-BF_4_ leads to the controlled formation of BF_3_ directly within the ionic liquid medium.^2^ This process generates a pre-electrolyzed anolyte containing a defined amount of BF_3_ (precisely tuned by the passing electric current), which can then be used as a catalytic system. Moreover, the intrinsic ionic nature of the solvent allows the avoidance of an additional supporting electrolyte, reducing waste and increasing the atom economy of the synthetic procedure. This methodology was successfully applied to a variety of transformations, including Friedel–Crafts/lactonization domino reactions, multicomponent condensations, Povarov reactions, and electrophilic aromatic substitutions. In many cases, the performance of the electrogenerated BF_3_ in ILs was comparable or superior to conventional Lewis acids (e.g., TiCl_4_, BF_3_·Et_2_O) while offering advantages in terms of safety, recyclability, and operational simplicity (Figure 2a–d, domino Friedel–Crafts lactonizations, Povarov reaction, Friedel–Crafts benzylation, and multicomponent reactions). For instance, the case of the Povarov reaction (Figure 2b), the classical chemical approach using etherate-BF_3_ led to only a 15% product yield, while applying the developed procedure dramatically increased the outcome to 96% yield. A key feature of this approach is also the recyclability of the ionic liquid, which can be reused after electrolysis, making the system attractive from a green chemistry perspective.

**Figure 2:**
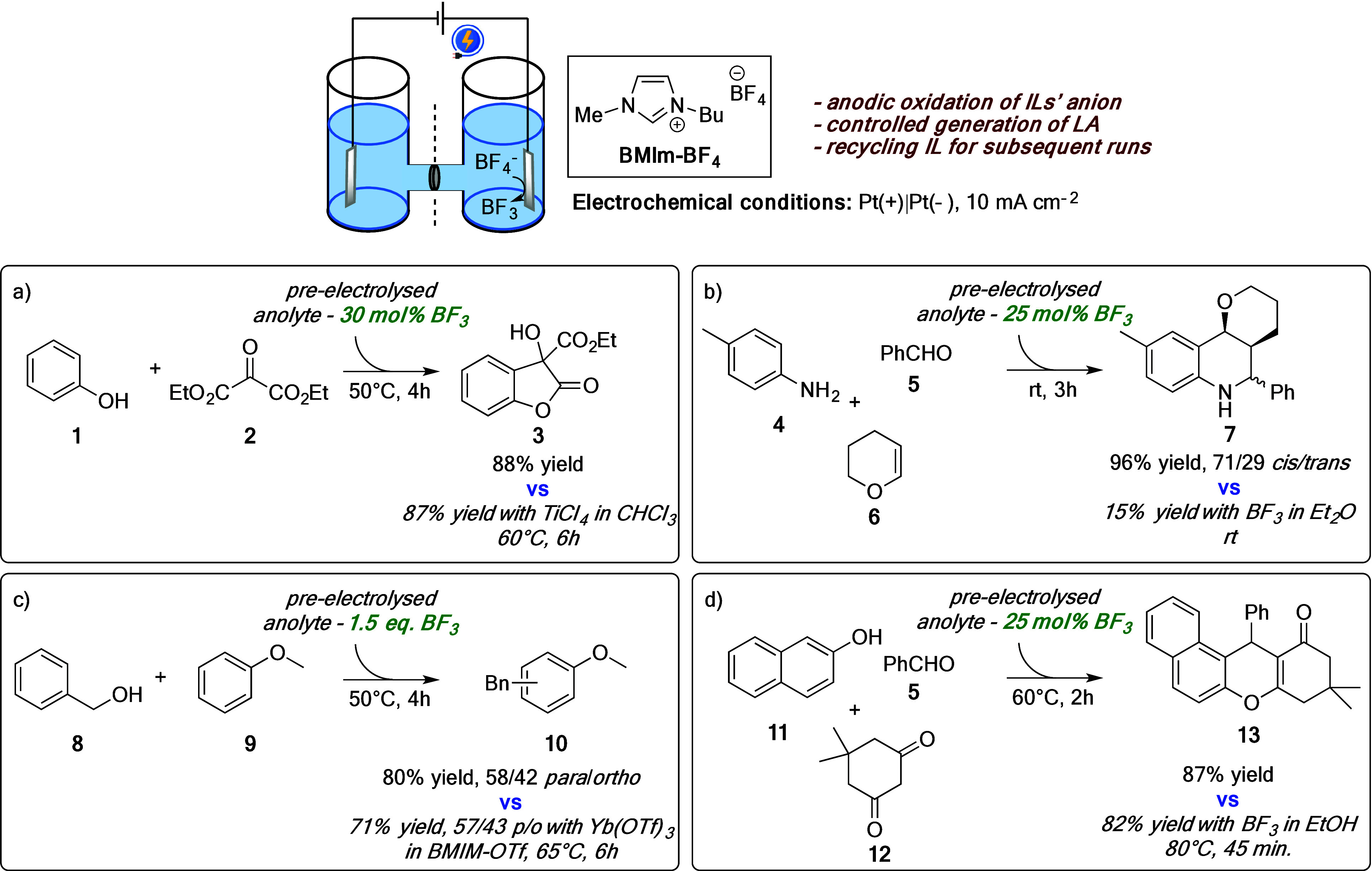
Electrogeneration of BF_3_. Literature data: (a) domino Friedel–Crafts lactonizations,^21^ (b) Povarov reaction,^22^ (c) Friedel–Crafts benzylation,^23^ and (d) multicomponent reaction.^24^

Subsequent theoretical and experimental investigations provided deeper insight into the nature of the active species generated in these systems.^1^ The reactivity of these electrogenerated systems was evaluated through the isomerization of styrene oxide (**14**), which served as a model reaction. Notably, the reaction outcome was strongly influenced by electrochemical conditions (divided vs undivided cells), highlighting the importance of controlling the speciation of active intermediates (Figure 3). As illustrated in Figure 3, the electrogeneration of BF_3_ in imidazolium-based ILs is accompanied by the formation of *N*-heterocyclic carbene (NHC) species, arising from cathodic processes. Within an undivided electrochemical cell setup, these NHCs can interact with BF_3_ to form adducts (NHC·BF_3_, Figure 3a, whose presence has been put in evidence by ^19^F NMR), which may serve as reservoirs or modulators of Lewis acidity. In fact, under these reaction conditions, the only reaction product formed was corresponding aldehyde **15**. Instead, if a divided cell setup was employed, dioxolane species **16** was obtained in 91% yield. The reason behind this difference in reactivity was explained via theoretical calculations. In fact, equilibria involving BF_3_ and BF_4_^–^ can lead to species such as B_2_F_7_^–^ (Figure 3b), which is formed predominantly in a divided electrochemical cell system, as supported by theoretical calculations. Additionally, the presence of BMIm^+^ further stabilizes the BF_3_ adduct, compared to the classic Et_2_O complex, without hindering its reactivity as the NHC. Hence, by simply switching the experimental setup, a divergent synthetic outcome can be achieved, in both cases ensuring a safer handling system compared to commercial BF_3_·Et_2_O. Finally, the structure of the IL cation was evaluated, leading to comparable excellent yields (up to 92%). These results demonstrate the promisingly broad applicability of different IL systems in various synthetic methods by replacing the ionic liquid imidazolium cation depending on the needed solvent properties (viscosity, solvation properties, and solubility of substrates) without interfering with LA electrogeneration. This study emphasizes that ionic liquids are not merely passive media but dynamic environments where multiple reactive species coexist, influencing both activity and selectivity.

**Figure 3:**
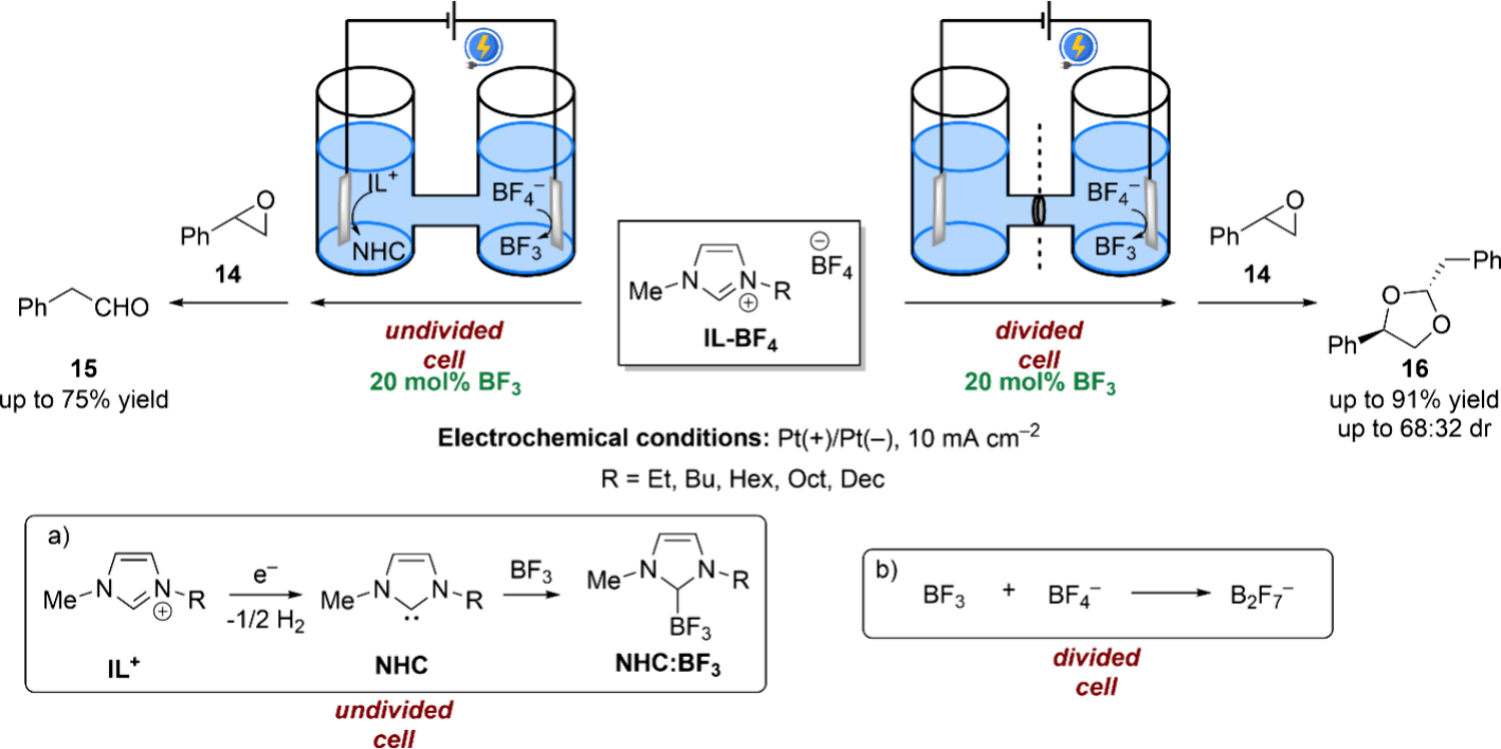
Styrene oxide isomerization via electrogenerated Lewis acids (EGLA): (a) formation of the NHC·BF_3_ adduct in undivided cells and (b) predominant formation of B_2_F_7_^–^ in divided cells.

Subsequently, another key contribution was reported, focusing on comparing the role of ionic liquids as (i) simple solvents for externally added BF_3_ and (ii) electroactive media capable of generating BF_3_ in situ as well as solvent and supporting electrolyte.^4^ Using the hydration of alkynes as a benchmark reaction (Figure 4), it was demonstrated that both approaches can deliver comparable yields (47–97% vs 51–94%, respectively). However, the electrochemical approach offers several advantages: (i) elimination of hazardous BF_3_ handling; (ii) tunable generation of the active species; and (iii) integration of solvent, catalyst, and supporting electrolyte in a single system.

**Figure 4:**
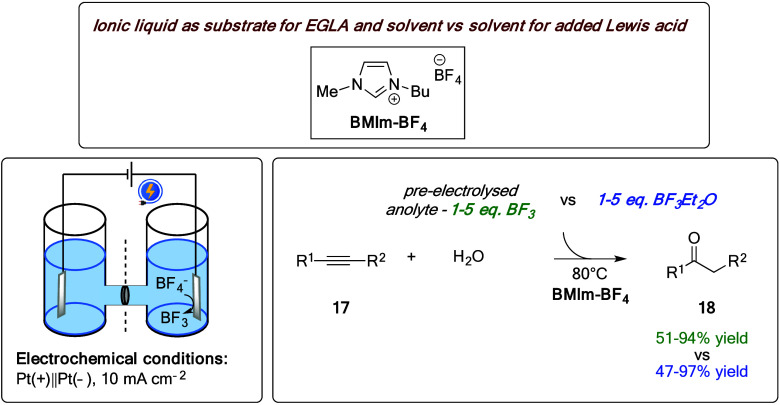
Hydration of alkynes: EGLA vs LA.

The evolution from classical Lewis acid chemistry in organic solvents to electrochemically driven systems in ionic liquids illustrates a clear trend toward more sustainable, controllable, and integrated catalytic platforms. Electrogeneration enables the on-demand formation of highly reactive species, while ionic liquids provide a unique environment that can stabilize, modulate, and even generate Lewis acids.

In particular, imidazolium-based ILs emerge as versatile electroactive media capable of bridging the gap between solvent and catalyst. The combination of electrochemistry and ionic liquids thus opens new avenues for designing greener and more efficient synthetic methodologies with fine control over reactivity and selectivity.

## Electroactive Ionic Liquids as NHC Precursors

Azolium cations are electroactive species: depending on their structure, they can be reduced at the cathode. When considering 1,3-disubstituted imidazolium cations, the reductive scission of the activated C–H bond (between the two nitrogen atoms) forms the corresponding *N*-heterocyclic carbene (NHC), whose fate depends on the reaction partners and solvent.^25^

This NHC (nucleophilic and basic) is usually highly reactive and unstable, rendering its isolation and characterization difficult. In classical organic chemistry, the formation of NHC from imidazolium salts is obtained by deprotonation with a strong base.

The presence of an NHC in an electrolyte can be put into evidence by voltammetric measurements from the corresponding oxidation peak.^26^

The cyclic voltammetry (CV) of a solution of a 1,3-disubstituted imidazolium salt shows in the forward cathodic scan a reduction peak at a potential dependent on the imidazolium structure, corresponding to the reductive C–H cleavage, followed by the presence of one or two anodic peaks on the reverse anodic scan. The voltammetric behavior of 1-butyl-3-methylimidazolium tetrafluoroborate (BMIm-BF_4_, one of the most used imidazolium salts) on a glassy carbon electrode is very similar in DMF and neat (Figure 5a^27^ and 5b,^28^ respectively); in both cases, the electrogenerated NHC presence is supported by the appearance of the anodic peak. A comparison between the anodic voltammetric behavior of BMIm-BF_4_ containing NHC (obtained by deprotonation with DBU, Figure 5c) and of electrolyzed BMIm-BF_4_ (Figure 5d) demonstrated the presence of NHC after cathodic reduction of the IL.^29^

**Figure 5:**
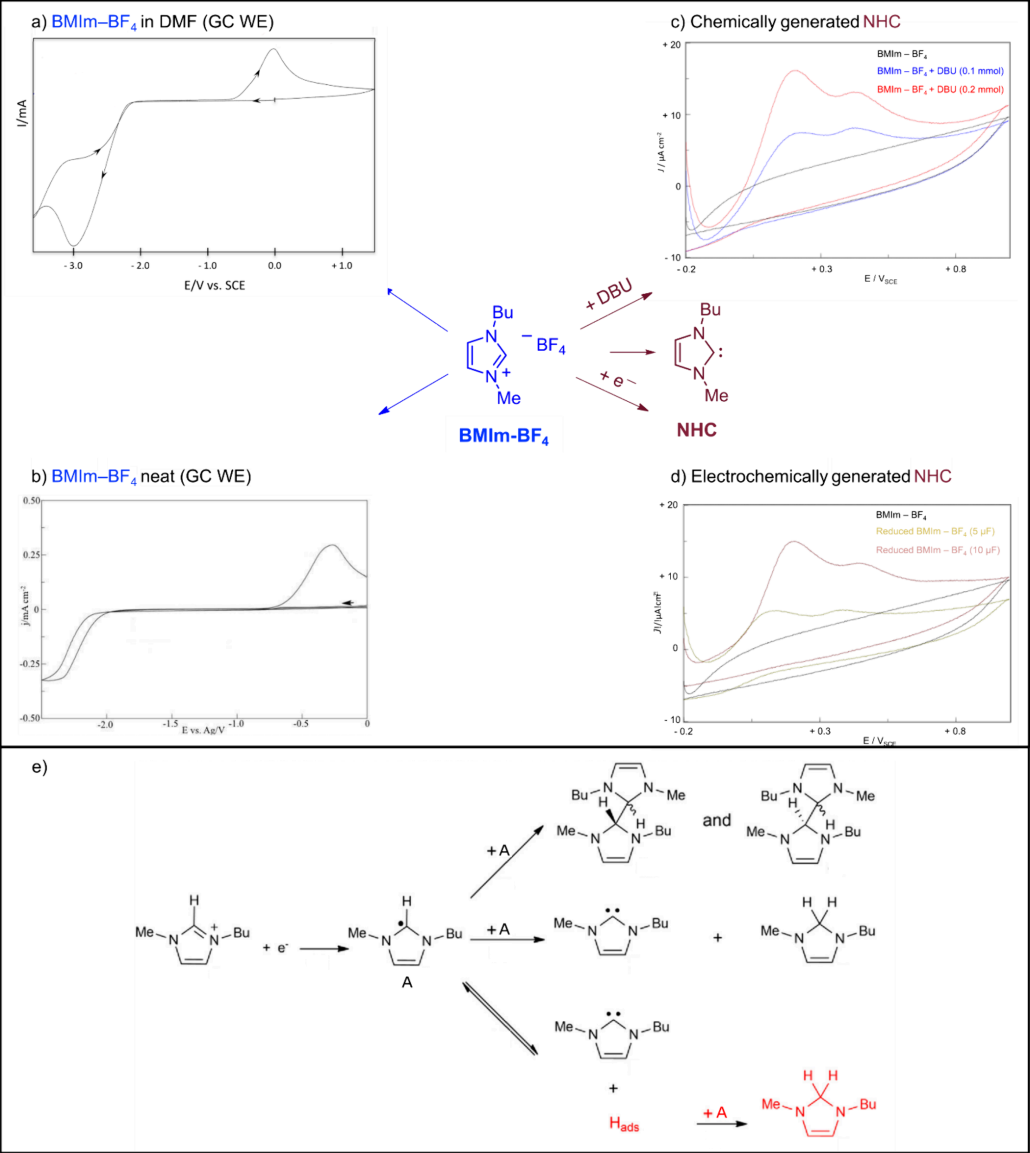
Voltammetric behavior of BMIm-BF_4_ on GC WE (a) in DMF and (b) neat. (c) Chemical and (d) electrochemical generation of NHC on Pt WE. (e) Proposed radical pathway on Au WE, adapted with permission from ref 32. Copyright 2018 Elsevier. CV data adapted with permission from (a) ref 27, copyright 2016 Wiley-VCH; (b) ref 28, copyright 2013 Elsevier; and (c, d) ref 29, copyright 2014 Wiley-VCH.

This behavior was first reported by Fuller and Carlin more than three decades ago^30^ in a THF solution of 1,3-bis­(4-methylphenyl)­imidazolium chloride. The authors did not attempt an interpretation of the CV, reporting that “the two oxidation waves seen during the reverse scan correspond to the generation of one or two electroactive species by the reduction process”.

More recently, Buess-Herman and co-workers verified, by NMR, the fate of NHCs electrogenerated on a gold electrode. Mostly dimeric products were obtained, along with reduced NHCs (Figure 5e).^31,32^

These products are suggested to originate mainly from a radical pathway, supported also by theoretical calculations.^33^ This radical pathway is in contrast with our results (*vide infra*) and with the electrosynthesis of imidazolium carboxylates by the reaction of electrogenerated NHC with CO_2_.^28,29,34,35^ Nonetheless, the different working electrodes (gold vs platinum) could explain the difference in behavior. In addition, Türkmen, Suzer, and co-workers were able to electrogenerate NHC in the XPS analysis chamber, giving direct spectroscopic evidence of NHC formation.^36^

The same behavior is found in dicationic ILs in which two imidazolium cations are linked by a spacer.^37^ The cathodic reduction of such species can yield a mono- or a di-NHC (actually both are obtained) whose molar ratio depends on the spacer nature (aliphatic or aromatic) and length (Figure 6a). Anyhow, it is possible to obtain NHCs following the same experimental procedure for monocationic imidazolium salts.^38^

**Figure 6:**
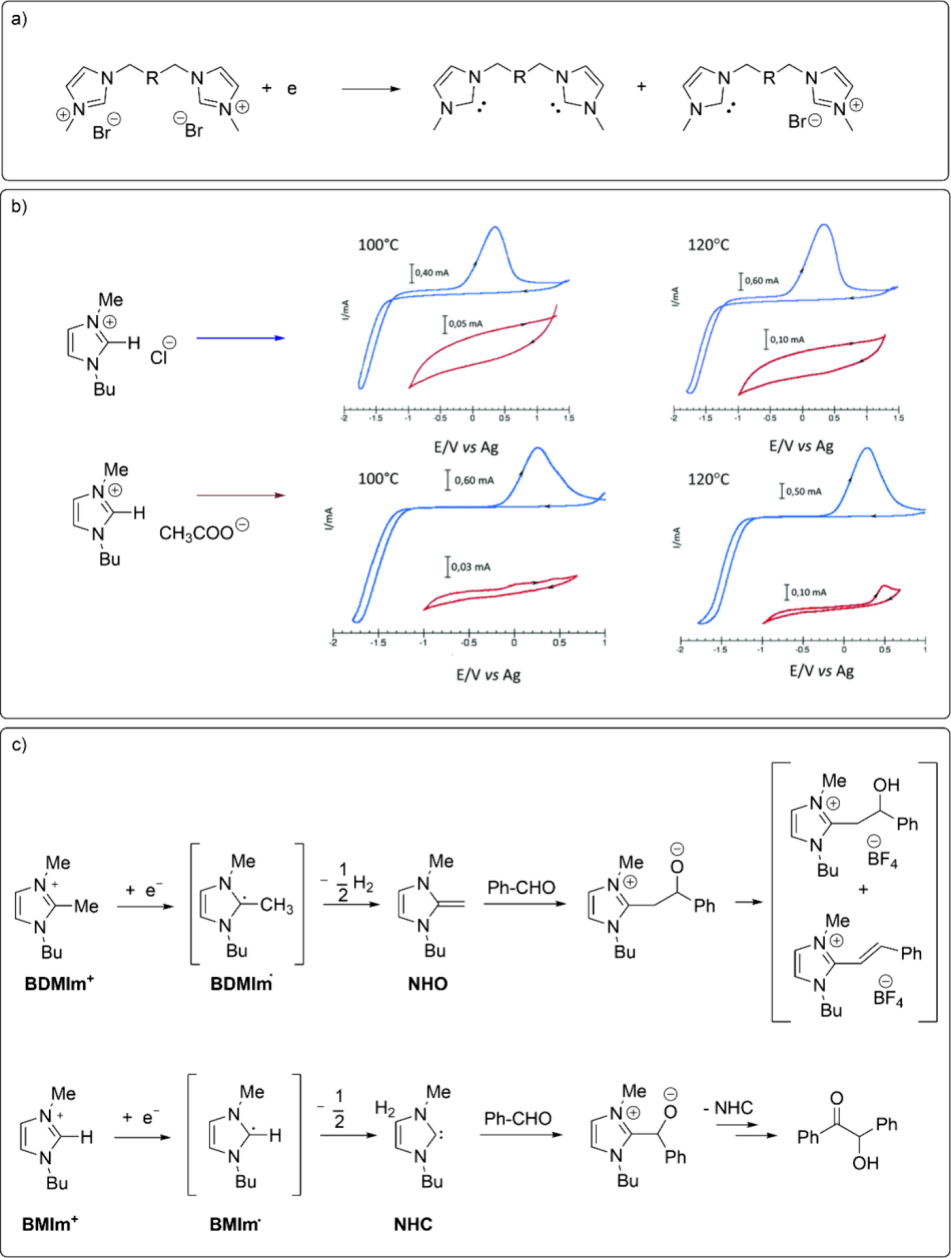
(a) Electrochemical behavior of dicationic ILs; (b) counteranion effect – generation of NHC via deprotonation by the acetate anion; and (c) BMIm^+^ and BDMIm^+^ electrochemical reduction and their reactivity toward aldehydes. CV data adapted with permission from ref 41. Copyright 2017 Royal Society of Chemistry.

The possibility to obtain NHC in IL without electrochemistry or strong bases was demonstrated by Nyulászi when studying the behavior of BMIm-Ac (1-butyl-3-methylimidazolium acetate), which they called “an organocatalytic ionic liquid”.^39^ In fact, the acetate ion basicity is sufficient to deprotonate to a low extent the imidazolium cation, generating small amounts of NHC able to catalyze the benzoin condensation. The voltammetric analysis again proves applicable for NHC presence determination in acetate IL. Despite the fact that the amount of NHC in BMIm-Ac at r.t. is so low that no oxidation current is seen in CV,^40^ when the temperature reaches 120 °C, an oxidation peak appears in the CV of pure BMIm-Ac,^41^ in contrast with the behavior of an imidazolium salt with a nonbasic counterion (BMIm-Cl) (Figure 6b).

Imidazolium cations are electroactive also if the 2-position is substituted by a methyl group. In this case, the cathodic event does not involve a ring carbon atom but rather the Cα-H bond. Consequently, an olefin is obtained, which is called NHO (*N*-heterocyclic olefin).^42^ Contrary to what happens with NHCs, an NHO has the highest electron density on the carbon outside the ring, which is the nucleophilic position. The addition of an NHO to the carbonyl of benzaldehyde is thus irreversible, yielding a stable adduct which does not evolve toward the release of NHO. As a consequence, NHO cannot be used as nucleophilic organocatalysts (Figure 6c).

Since NHC stability in solution strongly affects its catalytic ability, again voltammetric analyses can give evidence of the persistence of an NHC in solution. In fact, the electrochemical methodology generates NHCs only during the current flow, while classical chemical deprotonation continues to generate NHC until the complete consumption of the added base (usually present in a large excess). Our voltammetric studies demonstrated that the half-life of an electrogenerated carbene depends not only on the salt structure^27^ but also on the solvent^43^ and reaction partners.^44^

In particular, the ability of ILs to establish hydrogen bonds with NHCs stabilizes such species in pure ILs with respect to most organic solvents (e.g., DMF).^29,43^ Such experimental results were also supported by theoretical calculations.^27^

It is thus clear that NHC can be obtained from an imidazolium salt by cathodic deprotonation, despite the influence of the cathode nature (e*.*g., gold or platinum) on the imidazolium reduction pathway (yielding a radical or a carbene). This methodology is competitive with chemical deprotonation with two important advantages: first, the amount of electrogenerated carbene can be exactly controlled by the charge transferred to the imidazolium cation (i.e., NHC generation can be initiated or stopped by simply supplying or interrupting the current flow). This avoids the addition of bases and the generation of associated byproducts. The second advantage is that the use of a pure IL as an electrolyte not only circumvents the addition of a supporting electrolyte (one of the main criticisms to the electrochemical methodology in place of traditional chemical ones) but also enhances the stability of NHCs. The possibility of carrying out NHC generation in a divided electrochemical flow cell expands the advantages. In this way, a higher rate of NHC generation is possible with respect to batch electrochemistry, which is particularly significant in view of a possible process scale-up.^3^

It is our opinion that a deeper spectroscopic study would be beneficial in order to shed light on the anodic oxidation of NHCs, in which one or two current peaks are present, to define the products and possibly exploit such redox reactivity (which seems most probably not accessible by classical chemical means).

## Electrogenerated NHCs: Organocatalysis in Ionic Liquids

ILs are seldom considered good solvents for organocatalysis, including enantioselective methods. This is probably due to the fact that highly polar solvents could interfere with active catalytic intermediates. Nonetheless, there are some advantages in using ILs in catalysis: (a) water-sensitive catalysts are less sensitive to water in ILs due to the strong interactions between water and IL; (b) ILs stabilize polar transition states; (c) IL hydrogen bond acidity can influence reagents nucleophilicity, possibly enhancing selectivity; and (d) consequently, the selectivity and reaction rate can be different in ILs than in volatile organic solvents (VOCs).^45^ There are few literature reports concerning organocatalysis in ILs, one example being the enantioselective aldol reaction, successfully carried out in BMIm-BF_4_ by Jiang, obtaining high enantiomeric excesses (91 to 99% ee) in the reaction of both aromatic and aliphatic aldehydes with acetone with the use of an l-prolinamide derivative organocatalyst.^46^ Proline-derived organocatalysts are the preferred ones when using ILs as solvents, as reviewed by Zlotin,^47^ Šebesta,^11^ and Santos.^48^

With regard to imidazole-NHC in ILs, literature reports focus nearly exclusively on metal-NHC complexes in ILs (e.g., NHC-Pd-catalyzed oxyarylation of alkynes in imidazolium salts^49^) despite a few examples of the chemical generation of carbenes in ILs being reported.^50–52^ Although not explicitly cited in the relevant papers, each time that a base is used in an imidazolium IL, the intervention of the corresponding NHC cannot be ruled out.^53^ Most of the literature on the use of NHC in ILs deals with electrogenerated NHCs and their use as organocatalysts or bases, while their use as ligands in metal complexes is reported in VOCs.^54^ The different behavior of electrogenerated NHCs in ILs and VOCs, which is mainly due to the IL stabilization of polar compounds or intermediates, has been recently reviewed and is thus not covered here.^55^

The importance of NHCs as organocatalysts stems from their ability to give umpolung in carbonyl compounds (i.e., invert the carbonyl polarity) in which the carbon atom turns from electrophilic to nucleophilic (yielding the Breslow intermediate).^56–59^ This well-known chemistry is generally carried out in VOCs, using an excess of base to ensure the NHC’s continuous presence (by azolium salt deprotonation). When carrying out the electrogeneration of NHC in imidazolium ILs, the amount of NHC is easily controlled by switching the current flow on/off (without any added base and relative byproducts), and the NHC’s lifetime is usually enhanced (with respect to VOCs) due to the stabilization effect of ILs (*vide supra*). Moreover, ILs can stabilize polar intermediates, rendering this organocatalysis highly efficient. The first and most famous NHC-catalyzed reactions is the benzoin condensation, in which two molecules of benzaldehyde react to yield benzoin (2-hydroxy-1,2-diphenylethan-1-one, Figure 7a, blue product). When carried out by electrochemistry in BMIm-BF_4_, the reaction is efficient with a very broad scope and the catholyte (used IL in the cathodic compartment) can be recovered and recycled for subsequent runs, accentuating the sustainability profile of the strategy.^60,61^ In the presence of an alkylating agent and oxidant, electrogenerated NHC and benzaldehyde give an oxidative esterification (Figure 7a, red product). In this reaction, the NHC-aldehyde adduct (before the proton shift leading to the Breslow intermediate) is O-alkylated. Subsequent deprotonation and oxidation yield the corresponding ester.^62^

**Figure 7:**
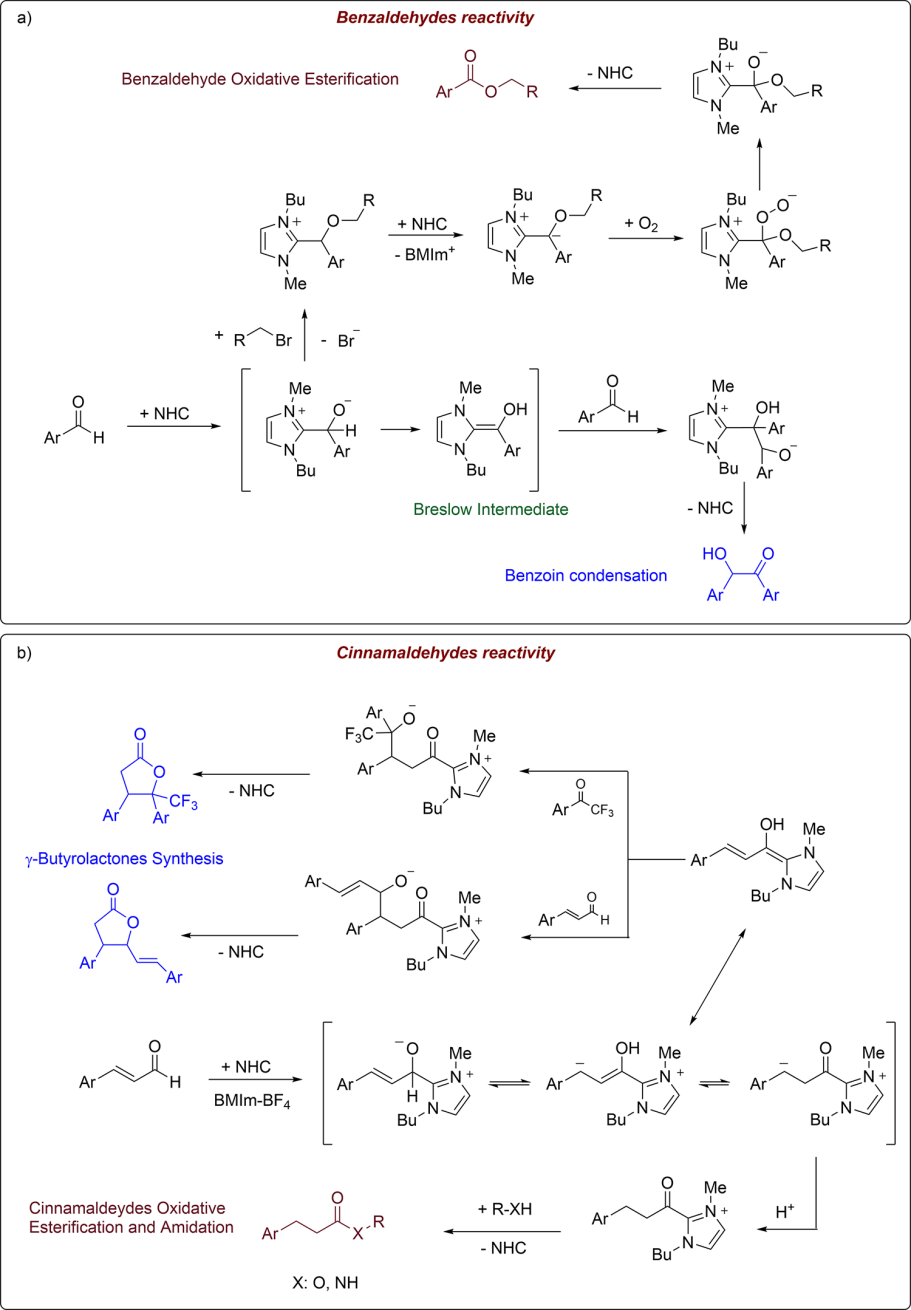
Organocatalytic synthetic applications of NHCs for oxidative transformations of (a) benzaldehyde derivatives or (b) cinnamaldehyde derivatives.

If the aldehyde has a conjugated C=C double bond (cinnamaldehyde derivatives, Figure 7b), then NHC reacts through a direct nucleophilic addition to the carbonyl group, finally evolving, in the presence of an alcohol or an amine, to the saturated ester^63^ or amide,^64^ respectively (Figure 7b, red product).

In the absence of any other reactant, cinnamaldehyde can dimerize to yield a γ-butyrolactone which still contains one of the two native double bonds, while in the presence of 2,2,2-trifluoromethyl­acetophenone the reaction yields a CF_3_-containing γ-butyrolactone (Figure 7b, blue products). In all cases, the catholyte could be recovered and reused up to 10 times, with only a moderate yield loss.^65^

The reaction of NHC, imine, and acid chloride yields a β-lactam via a Staudinger 2 + 2 reaction (Figure 8a), in which NHC is a nucleophilic catalyst, activating the imine nitrogen atom. The stereochemical outcome is a *cis*/*trans* β-lactam mixture, with a predominantly *trans* isomer (up to 9/91 *cis*/*trans*).^66,67^ β-Lactams can also be obtained in ILs with NHCs as bases, starting from a bromoamide. If a C3–C4 bond formation pathway is considered (Figure 8b), then a 2-bromoamide with suitable C–H acidity must be used (e.g., a malonate C–H group) to obtain an efficient deprotonation.^68^ On the other hand, when considering an N–C4 bond formation pathway, a 3-bromoamide is needed. In this case, two acidic positions are present, N–H and C2–H (Figure 8c), yielding a mixture of β-lactam and acrylanilide. When using NHC in IL as a base, a nearly 1:1 mixture of products is obtained, while when using a stronger electrogenerated base (DMF anion, obtained by deprotonation acted on by the product of the cathodic reduction of tetraalkylammonium cations) the selective formation of β-lactam is obtained.^69^ Besides the solvent effect (BMIm-BF_4_ vs DMF), the high base strength difference is decisive. In fact, the BMIm^+^ cation p*K*_a_ value is around 22, while the DMF one is around 27.

**Figure 8:**
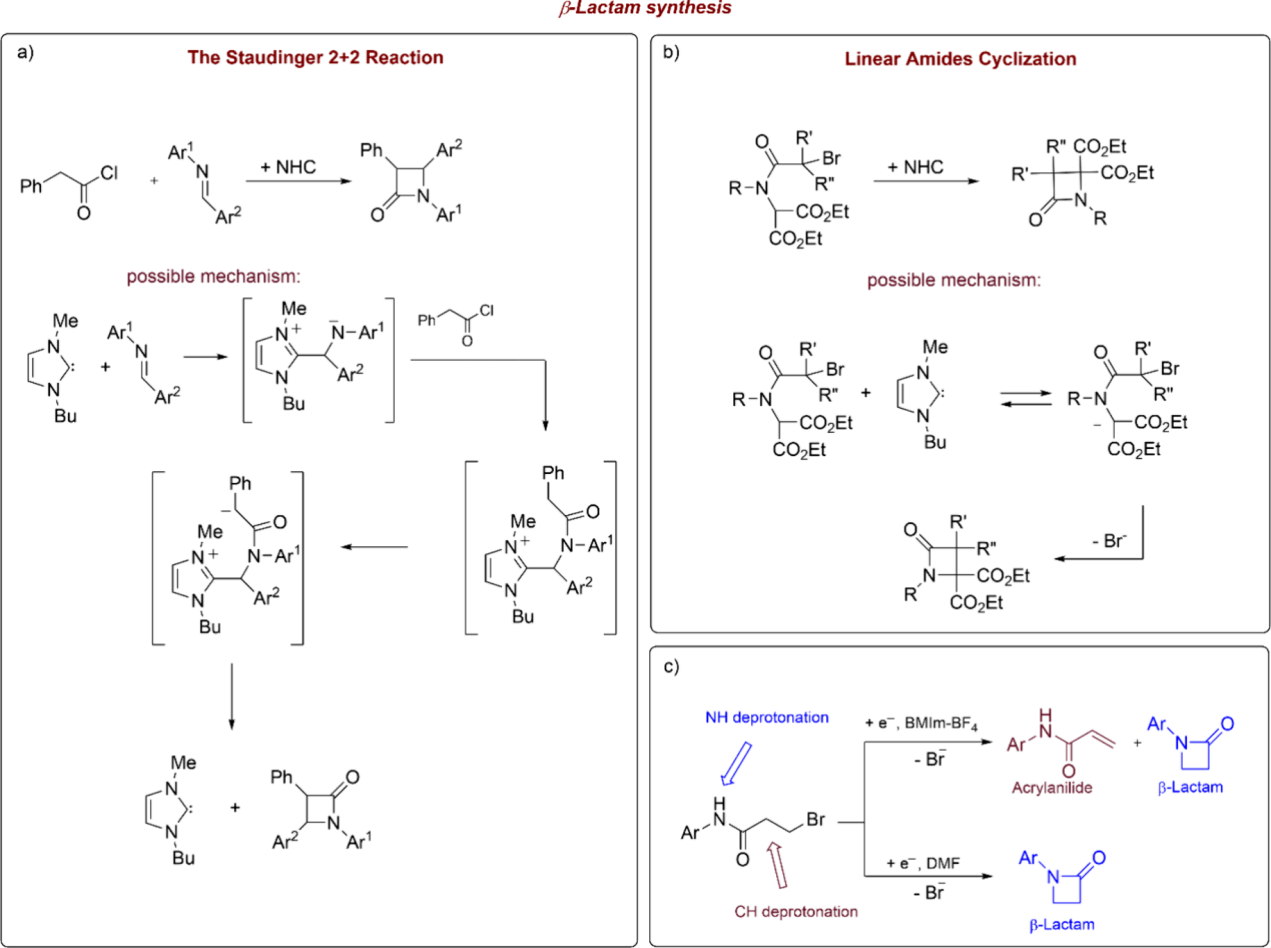
Synthesis of β-lactams via NHC catalysis: (a) the Staudinger reaction, (b) 2-bromoamide cyclization, and (c) selectivity in β-lactam formation with electrogenerated bases.

These examples show that NHC electrogeneration in ILs can be an efficient method to promote organocatalytic reactions. Moreover, the fact that electrochemistry allows us to avoid bases and related byproduct formation and the possibility to recover the catholyte and reuse it in subsequent runs (up to 10) are certainly bright sides from the perspective of green chemistry.

NHCs’ basic and nucleophilic character could create a competition between two different reactivities or establish a synergy, as in the case of benzaldehyde oxidative esterification. ILs’ p*K*_a_ values place a limit on the strength of the bases that can be effectively used in their combination, so if a stronger base than NHC is needed, a change in solvent is necessary.

## Electrogenerated NHCs in Ionic Liquids: The Storage Possibility

As previously stated, NHCs are highly reactive species, although stabilized in ILs. This implies that their generation must be carried out just prior to their use. Nonetheless, the high NHC nucleophilicity allows a rapid reaction with CO_2_, forming a stable NHC–CO_2_ adduct. Such an adduct can be regarded as an NHC reservoir, whose release can be obtained by heating (*T* > 100 °C) or ultrasound irradiation.^34^

The efficiency of such NHC release was demonstrated in the previously described benzoin reaction and cinnamaldehyde oxidative esterification (Figure 9a), obtaining comparable yields to freshly prepared NHC.^28^ Moreover, a tentative released NHC quantification was probed by reaction with elemental sulfur, yielding the corresponding imidazole-2-thione (Figure 9b).^70^ After 3 days (in air) from electrogenerated NHC–CO_2_ adduct formation in IL, the thione yield was still quite high (87 vs 99% with freshly prepared NHC). It should be underlined that, despite the IL “protecting” effect toward NHCs, if the same delayed reaction is carried out on a solution of NHC in IL, the thione yield is as low as 28% (Figure 9b).

**Figure 9:**
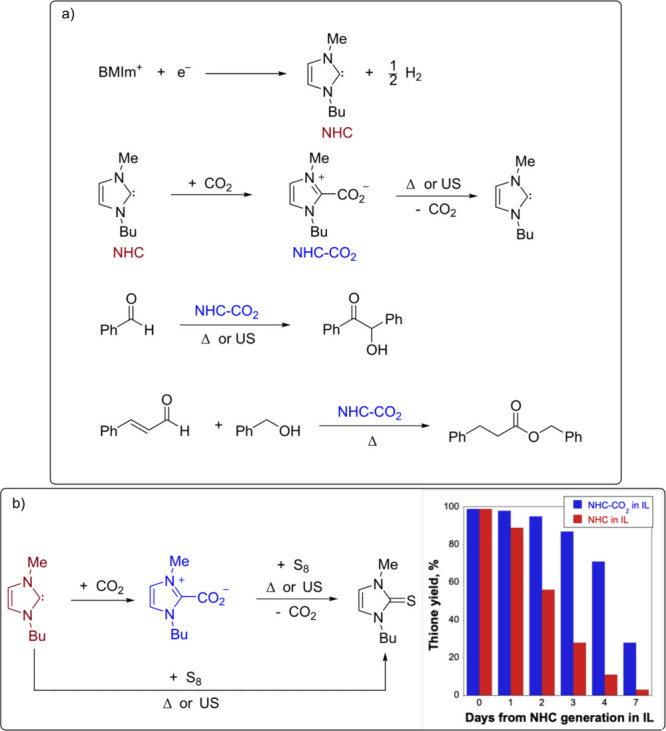
(a) Stabilization and release of NHCs-CO_2_ and (b) reaction with elemental sulfur.

It is thus clear that the combination of electrochemical NHC generation, NHC protecting effect of IL as solvent, and reversible NHC–CO_2_ adduct formation enables the expansion of NHC use beyond the VOC boundaries.

## Conclusions

The combination of electrochemistry and ILs constitutes a powerful tool for organic reactions in a greener way. In particular, the possibility to inject energy into the chemical system in the form of electricity (potentially from renewable sources) allows for redox reactions without the need for stoichiometric redox agents. Moreover, the simple regulation or interruption of the current flow permits precise dosing of electroactivated reagents. The major criticisms leveled at this technique (specialized equipment and large amounts of supporting electrolyte) have been overcome by the advent of commercial, cheap, and easy-to-handle equipment and by the possibility to use ionic species (ILs) as both solvent and supporting electrolyte. Their electrochemical window (the potential interval in which the electrolyte itself is not electroactive) can be seen as a measure of the involvement of the electrolyte in the electrode processes. Usually, such an involvement is avoided, but in some cases, this electroactivity can be an advantage. In particular, imidazolium tetrafluoroborate ILs can electrogenerate NHC at the cathode and BF_3_ at the anode. The main advantage of such processes (besides those previously mentioned) is that the IL environment stabilizes such highly reactive species, thus modulating their reactivity. On the other hand, the high IL viscosity and hygroscopicity could be regarded as impediments to the performance of these reactions. While this is certainly true for viscosity (sometimes requiring high temperatures), it is not entirely accurate for hygroscopicity. Indeed, water is highly segregated in ILs (sometimes used as dehydrating agents), and usually, a minor sensitivity to water is experienced in ILs. IL electroactivation, far from being the solution to all problems regarding NHC- and BF_3_-catalyzed reactions, should be considered as an opportunity in organic synthesis. Moreover, the demonstrated possibility to reuse the electrolyte in subsequent runs places ILs within the scope of green chemistry. In conclusion, our suggestion to new organic chemist generations is to keep an open mind about electrochemistry, which can allow an expansion of their possibilities beyond the boundaries of classical organic methodologies and solvents.
